# Neural machine translation of chemical nomenclature between English and Chinese

**DOI:** 10.1186/s13321-020-00457-0

**Published:** 2020-08-31

**Authors:** Tingjun Xu, Weiming Chen, Junhong Zhou, Jingfang Dai, Yingyong Li, Yingli Zhao

**Affiliations:** Shanghai Institute of Organic Chemistry, Chinese Academy of Sciences, 345 LingLing Road, Shanghai, 200032 China

## Abstract

Machine translation of chemical nomenclature has considerable application prospect in chemical text data processing between languages. However, rule based machine translation tools have to face significant complication in rule sets building, especially in translation of chemical names between English and Chinese, which are the two most used languages of chemical nomenclature in the world. We applied two types of neural networks in the task of chemical nomenclature translation between English and Chinese, and made a comparison with an existing rule based machine translation tool. The result shows that deep learning based approaches have a great chance to precede rule based translation tools in machine translation of chemical nomenclature between English and Chinese.

## Introduction

Chemical names are primitive representations of chemicals, widely used by chemists in research articles, patents, data materials to describe chemical substances. Names accorded with chemical nomenclatures of IUPAC and CAS are exact expressions of molecular structures [1, 2], therefore those names can be used as identifiers of substances in chemical databases, and can be recognized by machine easily for converting names to conventional chemical structure representations [3, 4]. English and Chinese are the two most used languages of chemical nomenclature in the world, according to number of results found by Google for searching a chemical name in different languages [5]. However, the linguistic differences of English and Chinese chemical names have limited exchanges between users on both sides [6]. Therefore, machine translation of chemical nomenclature would be more applicable than manual translation in chemical data processing. For example, data sets of compound names would be more valuable when derived from chemical named entity recognition systems of Chinese text-mining materials, because bulk translation of Chinese chemical names into English by machine make it possible for the names to be converted into connection tables of chemical structures, owing to the fact that the vast majority of “name to structure” tools are only for English nomenclature [7–9].

Unfortunately, it still has a lot of work to be done in machine translation of chemical nomenclature beyond existing researches [10–12], especially in translation of chemical names between English and Chinese [13, 14]. There has significant complication in the task of analyzing Chinese chemical names, and make it difficult to set up a sophisticated machine translation rule set for conversion of various Chinese chemical names to or from English [13, 14]. For example, the Chinese chemical name of “ethyl acetate” is “乙酸乙酯”, there is not only no word boundaries (spaces) in Chinese chemical names, but the order of words is also reversed, “ethyl” for “乙酯”, “acetate” for “乙酸”, and there is a special case of “ethyl” translated into “乙酯”, because it would be “乙基” or “乙” in other names such as “ethyl alcohol” (乙醇). The word “ol” of hydroxyl in English organic compound names is often confused in Chinese names, it would be “醇” when the OH group bond with an aliphatic parent, for example “methanol” is translated into “甲醇”, but it would be “酚” when the OH group bond with an aromatic ring, for example “benzene-1,2,4-triol” is translated into “苯-1,2,4-三酚”. Similar cases exist widely in translation of chemical nomenclature between English and Chinese.

Therefore, rule based machine translation of chemical nomenclature between English and Chinese requires specialized expertise, and need a formally trained chemist who is fluent in English and Chinese languages to build a perfect set of rules [8, 13, 14]. Machine Learning (ML) based approaches may be more applicable for the task of chemical nomenclature translation, because there is no need for building complex rule set, and in view of that there already have various applications in the field of neural machine translation [15–17]. We herein describe two Deep Learning (DL) based approaches of neural machine translation of chemical nomenclature between English and Chinese, and make a comparison with an existing rule based machine translation tool.

## Materials and methods

ML based approaches ideally require large data sets from which to learn, and quality of the data is critical. Obtaining such data set of chemical names is a significant challenge, as many are maintaining in one language (English or Chinese), and others are below-standard quality for ML use. Furthermore, translation of chemical nomenclature between English and Chinese is not inherent symmetry, that means an English chemical name have a translation of Chinese name which may be not suitable for back-translation. For example, “β-phenethylol” is translated into “β-苯乙醇” in Chinese, but back-translation of “β-phenylethyl alcohol” seems more appropriate. That is to say, the reversed version of training data for English to Chinese translation model may be not suitable for Chinese to English translation model.

Therefore, we built data sets on the basis of our chemical data analyzing system, the system has a corpus manually curated from scientific literatures and compiled by our data analysts. The corpus includes chemical names and their manual translations (English and Chinese), and the names are various from systematic compound names to trivial names. We extracted chemical names and their translations from the corpus, and made up two data sets, English names translated into Chinese names (En2Ch) batch and Chinese names translated into English names (Ch2En) batch. For names that have multiple translations, we chose the translations of chemical names that most used by our analysts in the data analyzing system. Eventually, we obtained the data sets of 30,394 records for En2Ch and 37,207 records for Ch2En both duplicate removed, and each data set is made up of source names and target names in two languages (Additional file 1). The data set has 100 unique characters in English name strings, and 2,056 unique characters in Chinese translated name strings.

**Fig. 1:**
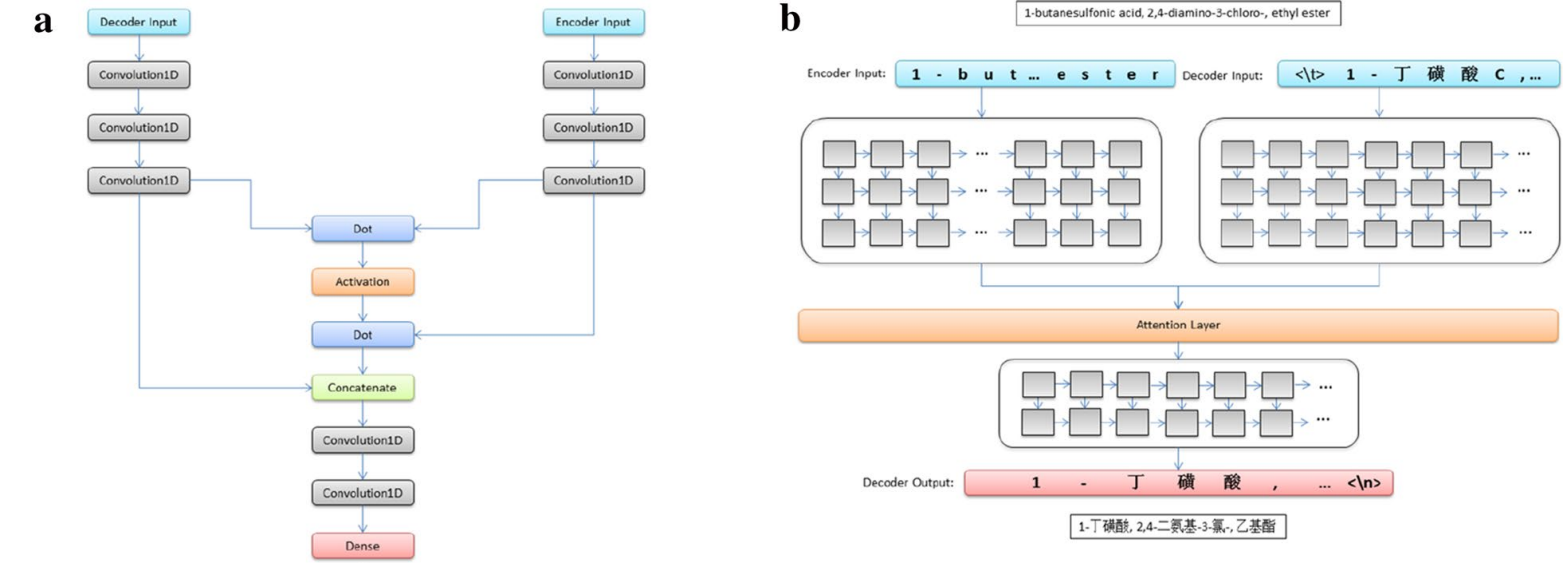
Architecture of the CNN based neural networks for machine translation of chemical nomenclature (**a**). Illustration of the CNN based neural networks for machine translation of chemical nomenclature in training mode (**b**)

We first applied a character-level sequence to sequence Convolutional Neural Network (CNN) based neural networks for translation of chemical nomenclature [18–20]. The neural networks conceptually consists of four elements: An encoder of three one-dimensional CNN layers encodes the input character sequence; A decoder of three one-dimensional CNN layers turns the target sequences into the same sequence but offset by one timestep in the future; Attention mechanism layers take outputs of the encoder and decoder; And a decoder of two one-dimensional CNN layers decodes the output character sequence, as shown in Fig. 1. The input chemical name strings are transformed into embedding sets of vectors. The number of vectors equals the number of unique characters in all input chemical names, and provided as an input to the encoder–decoder model with attention mechanism. The output strings are reversed from predicted sequences by re-embedding.

**Fig. 2:**
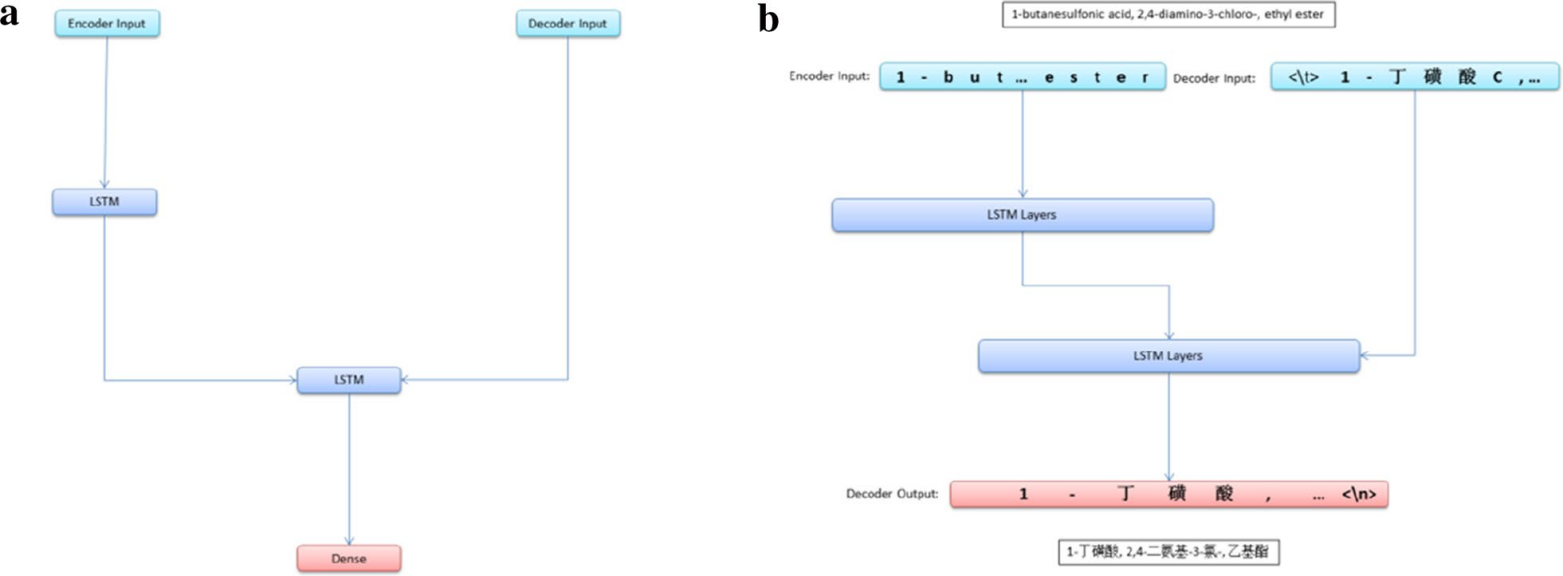
Architecture of the LSTM based neural networks for machine translation of chemical nomenclature (**a**). Illustration of the LSTM based neural networks for machine translation of chemical nomenclature in training mode (**b**)

Recurrent Neural Networks (RNNs) and specifically Long Short Term Memory (LSTM), are also deep learning architectures well suited to the translation of variable-length sequences [15, 21, 22]. We then applied a LSTM based neural networks for translation of chemical nomenclature [15, 18, 23]. The LSTM based neural networks have an encoder of LSTM layers, the encoder turns input sequence to 2 state vectors, and a decoder of LSTM layers is trained to turn the target sequences into the same sequence but offset by one timestep in the future, and the decoder uses the state vectors from the encoder as initial state, this is a process called "teacher forcing", as shown in Fig. 2.

The neural networks were implemented in Python 3.7 [24] using Keras 2.3 [25] and TensorFlow backend [26]. The neural network models were trained on each data set of En2Ch and Ch2En. We split the data set for cross-validation at random, 80% for training set and 20% for validation set. The parameters of the neural networks were chosen according to the performances on the validation set: Batch size for training is 64, number of epochs is 100, latent dimensionality of the encoding and decoding space is 256 in CNN (Additional file 2) and LSTM (Additional file 3) based neural networks.

**Fig. 3:**
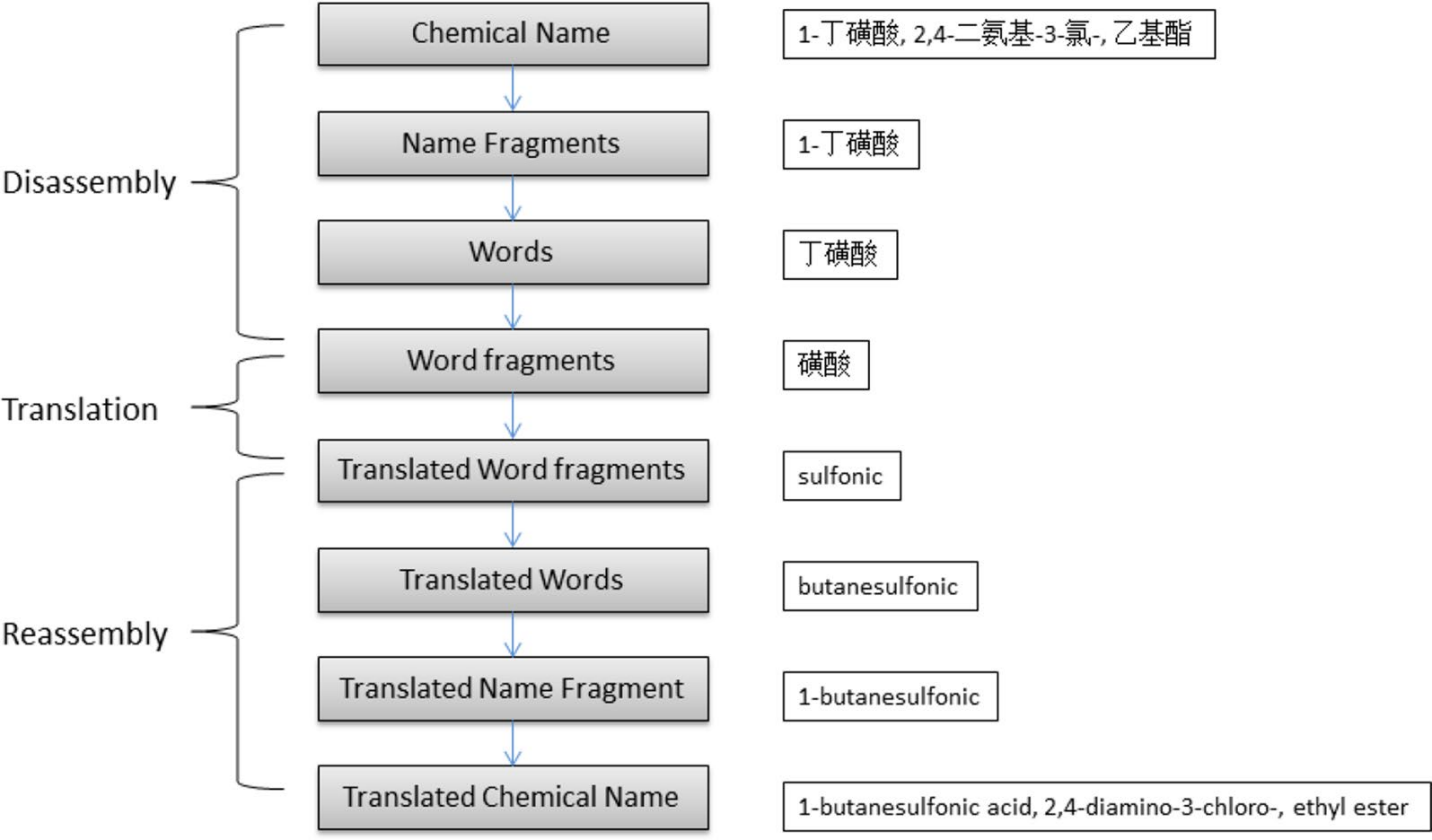
Illustration of the rule based machine translation tool of chemical nomenclature

For the purpose of contrast analysis between DL based and rule based machine translation of chemical nomenclature, we used an existing rule based machine translation tool [27] on same data sets of the neural network models. Processes of the translation tool are conceptually consists of three steps: Disassembly, translation and reassembly. In a procedure of translation, a chemical name will be analyzed by the translation tool first, disassembled to word fragments, translated into target language, and then reassembled to a translated chemical name, all the processes are in compliance with a rule set, as shown in Fig. 3.

## Results and discussion

We toke the same testing data from the data set for DL and rule based machine translation of chemical nomenclature, 6,079 data records of English names translated into Chinese names (En2Ch), and 7,441 data records of Chinese names translated into English names (Ch2En). The results (Additional file 4) were analyzed in five fields as shown in Table 1, each field has values of performance at En2Ch and Ch2En. Success Rate: Percentage of translated names that each approaches successfully produced; String Matching Accuracy: Exactly string matching accuracy of translated names from each approaches and target names from the data sets; Data Matching Accuracy: Exactly string matching with data of translated names from the corpus which has multiple translations of one chemical name; Manual Spot Check: Manual spot check accuracy of 100 random names from the testing data sets for each result, and the checker was "blind" for which system generated the results; Running Time: Time of each approach running through the testing data sets in the same computing environment.

**Table 1:** The performances and comparisons between DL and rule based machine translation of chemical nomenclature

Field	CNN based	LSTM based	Rule based
Success Rate En2Ch	100%	100%	75.97%
Success Rate Ch2En	100%	100%	59.90%
String Matching Accuracy En2Ch	82.92%	89.64%	39.81%
String Matching Accuracy Ch2En	78.11%	55.44%	43.77%
Data Matching Accuracy En2Ch	84.44%	90.82%	45.15%
Data Matching Accuracy Ch2En	80.22%	57.40%	44.91%
Manual Spot Check En2Ch	90.00%	89.00%	80.00%
Manual Spot Check Ch2En	82.00%	61.00%	78.00%
Running Time En2Ch (s)	1423	190	288
Running Time Ch2En (s)	1876	303	322

The two DL based approaches (CNN and LSTM) successfully produced translated names of all the testing data, owing to that the neural network models can always give an output unless there are characters absent from the training data [28]. However, the rule based translation tool failed in producing translated names at a considerable proportion, especially on the Ch2En data set. For example, the translation of “羽扇豆醇棕榈酸酯”, “异氧化前胡素”, “原儿茶酸甲酯”, etc. We believe failures in the processes of name disassembly and word fragments translation caused interrupts of the program, especially because no word boundaries (spaces) in these Chinese chemical names, leads to word segmentation errors, commonly happened in translation of long trivial names. Most trivial names of natural products are usually derived from the species names of their biological sources [29, 30], translation of these names may be irregular [6], some Chinese natural product names are even transliterations, and lead to failures of the rule based translation tool.

The performances of each approach at data matching with translated names from the corpus are all better than string matching with target names from the data set, for the reason that chemical names usually have more than one translation in the corpus. The two DL based approaches seem much more qualified for translation of chemical nomenclature, as observed from the result data, except the Ch2En batch produced by LSTM based neural networks. Although LSTM neural networks have fixed vanishing gradient problem of traditional RNNs [31], it seems still have deficiencies in learning long-term dependencies of Chinese characters, seeing that a lot of false results translated by the LSTM based neural networks appear at the end of chemical names, for example, “7-十八烯酸甲酯” (7-octadecenoic acid, methyl ester) was translated into “7-oleic acid, diethyl ester”, “3-甲氧基苯乙酸” (3-methoxyphenylacetic acid) was translated into “3-methoxy cinnamal”, etc.

Manual spot check (Additional file 5) shows that performances presented by the two DL based approaches are close to string matching accuracy, but there is a large gap between performances presented by the rule based translation tool. We believe the main reason is multiple translations of one chemical name, but rule based translation tools are always constrained by relatively fixed rule sets, therefore the names produced by translation tools may not match with target names but they are also appropriate. For example, “p-toluene” (对甲苯) was translated into “p-甲苯”, “ethenyl hexanoate” (己酸乙烯基酯) was translated into “己酸乙烯酯”, etc.

The CNN based approach cost significantly more time than the other two, it could be the complexity of neural network architecture that decides computing cost of running through the testing data sets. We also found that some names translated by the two DL based approaches are better than target names in the data set. For example, the target name of “1-methoxy-4-methyl-benzene” is “对甲基苯甲醚” in the data set of En2Ch batch, although the target name is an alternative representation of this specific compound, it is better to be translated into “1-甲氧基-4-甲基-苯” literally. That is probably one of the reasons why the manual spot check accuracy of the two DL based approaches are all slightly better than matching accuracy.

We picked out 100 target names using IUPAC and CAS naming systems from Ch2En batch for manual check, to find out how different naming systems affect the translation. The result (Additional file 6) listed in Table 2 shows that the performances presented by rule based approach are close, but the performances presented by the two DL based approaches are better on IUPAC names than CAS names. We also evaluated the three approaches when translate chemical names of different length in Ch2En batch, we took the average length of 6 (Chinese characters) as a line of demarcation, as shown in Table 2. The rule based approach did not seem to be affected by the length of chemical names, but may be more applicable for regular names, because there are more systematic names in long chemical names. The neural networks we applied here are more applicable for translation of short sentences, seeing that the two DL based approaches made better performances on short chemical names.

**Table 2:** The performances and comparisons of translating chemical names using different naming systems and having different length

Field	CNN based (%)	LSTM based (%)	Rule based (%)
IUPAC names	92.00	62.00	80.00
CAS names	80.00	52.00	78.00
Length not greater than 6	80.42	60.47	38.81
Length greater than 6	74.38	47.31	49.33

## Conclusion

After comparison between two neural machine translation approaches and one existing rule based translation tool, we found that DL based approaches may precede rule based translation tools in general, but DL based approaches highly depend on quality and quantity of training data, and rule based tools highly depend on perfection of rule sets. We can not guarantee correctness of all chemical names translated by the two types of neural networks, but they had showed high accuracy. However, the rule based translation tool made much lower success rate, but it had considerable accuracy in manual spot check too. Further more, combination of the two types of neural networks (CNN and LSTM) may have greater capability [22, 32, 33], and would improve performance of LSTM based neural networks on Chinese chemical name translation.

One of the most common applications for Chinese chemical names translating into English is in scientific publications, because most of chemical journals in China request authors to provide abstracts both in Chinese and English, and some of editors require English chemical names in main text of manuscripts. Moreover, the rule based translation tool we applied here has been used as online services more than one million times in recent years [27]. Therefore, we believe the neural machine translation of chemical nomenclature we studied has considerable application prospect, and can provide new solutions not only for chemical data processing but for common use as well.

## Supplementary information

**Additional file 1:** Training data set.

**Additional file 2:** Python code for CNN model.

**Additional file 3:** Python code for LSTM model.

**Additional file 4:** Testing and result data set.

**Additional file 5:** Manual spot check and result data set.

**Additional file 6:** Manual check for translation of chemical names using different naming systems.

## Data Availability

The data set for training and validation, the python code for generating the neural network models, and the result data set are included in the Additional files.

## Abbreviations

ML: Machine learning
DL: Deep learning
CAS: Chemical Abstracts Service
CNN: Convolutional neural network
RNN: Recurrent Neural Network
LSTM: Long Short Term Memory
IUPAC: International Union of Pure and Applied Chemistry
En2Ch: English chemical names translated into Chinese chemical names
Ch2En: Chinese chemical names translated into English chemical names
